# Repertoire-based mapping and time-tracking of T helper cell subsets in scRNA-Seq

**DOI:** 10.3389/fimmu.2025.1536302

**Published:** 2025-04-04

**Authors:** Daniil K. Lukyanov, Valeriia V. Kriukova, Kristin Ladell, Irina A. Shagina, Dmitry B. Staroverov, Bella E. Minasian, Anna S. Fedosova, Pavel Shelyakin, Oleg N. Suchalko, Alexander Y. Komkov, Konstantin A. Blagodatskikh, Kelly L. Miners, Olga V. Britanova, Andre Franke, David A. Price, Dmitry M. Chudakov

**Affiliations:** ^1^ Center for Molecular and Cellular Biology, Moscow, Russia; ^2^ Genomics of Adaptive Immunity Department, Institute of Bioorganic Chemistry, Moscow, Russia; ^3^ Institute of Clinical Molecular Biology, Kiel University, Kiel, Germany; ^4^ Division of Infection and Immunity, Cardiff University School of Medicine, University Hospital of Wales, Cardiff, United Kingdom; ^5^ Institute of Translational Medicine, Pirogov Russian National Research Medical University, Moscow, Russia; ^6^ Abu Dhabi Stem Cell Center, Al Muntazah, United Arab Emirates; ^7^ Systems Immunity Research Institute, Cardiff University School of Medicine, University Hospital of Wales, Cardiff, United Kingdom; ^8^ Department of Molecular Medicine, Central European Institute of Technology, Brno, Czechia

**Keywords:** helper T cell subsets, scRNA-Seq, scTCR-seq, immune repertoires, T cell memory, Th22, Th17, cytotoxic CD4+ T cells

## Abstract

**Introduction:**

The functional programs of CD4^+^ T helper (Th) cell clones play a central role in shaping immune responses to different challenges. While advances in single-cell RNA sequencing (scRNA-Seq) have significantly improved our understanding of the diversity of Th cells, the relationship between scRNA-Seq clusters and the traditionally characterized Th subsets remains ambiguous.

**Methods:**

In this study, we introduce TCR-Track, a method leveraging immune repertoire data to map phenotypically sorted Th subsets onto scRNA-Seq profiles.

**Results and discussion:**

This approach accurately positions the Th1, Th1-17, Th17, Th22, Th2a, Th2, T follicular helper (Tfh), and regulatory T-cell (Treg) subsets, outperforming mapping based on CITE-Seq. Remarkably, the mapping is tightly focused on specific scRNA-Seq clusters, despite 4-year interval between subset sorting and the effector CD4^+^ scRNA-Seq experiment. These findings highlight the intrinsic program stability of Th clones circulating in peripheral blood. Repertoire overlap analysis at the scRNA-Seq level confirms that the circulating Th1, Th2, Th2a, Th17, Th22, and Treg subsets are clonally independent. However, a significant clonal overlap between the Th1 and cytotoxic CD4^+^ T-cell clusters suggests that cytotoxic CD4^+^ T cells differentiate from Th1 clones. In addition, this study resolves a longstanding ambiguity: we demonstrate that, while CCR10+ Th cells align with a specific Th22 scRNA-Seq cluster, CCR10−CCR6+CXCR3−CCR4+ cells, typically classified as Th17, represent a mixture of bona fide Th17 cells and clonally unrelated CCR10^low^ Th22 cells. The clear distinction between the Th17 and Th22 subsets should influence the development of vaccine- and T-cell-based therapies. Furthermore, we show that severe acute SARS-CoV-2 infection induces systemic type 1 interferon (IFN) activation of naive Th cells. An increased proportion of effector IFN-induced Th cells is associated with a moderate course of the disease but remains low in critical COVID-19 cases. Using integrated scRNA-Seq, TCR-Track, and CITE-Seq data from 122 donors, we provide a comprehensive Th scRNA-Seq reference that should facilitate further investigation of Th subsets in fundamental and clinical studies.

## Introduction

Clonal populations of CD4^+^ T helper (Th) cells orchestrate the course of an immune response via specific interactions with peptide epitopes presented in complex with major histocompatibility class II (MHCII) molecules. Their functional and antigen-specific diversity allows them to guide both classical (i.e., B cells, dendritic cells, and macrophages) and non-classical (i.e., endothelial cells, epithelial cells, and granulocytes) antigen-presenting cells to optimize effector functionality (1–3). Inappropriate Th responses to certain antigens have been associated with an impaired pathogen clearance (4–6); inefficient response to vaccination (7); acute and chronic hypersensitivity, inflammation, and inflammaging (8–11); autoimmunity (12–15); and cancer (16). Accordingly, when investigating T-cell responses, it is critical not only to quantify the magnitude of the antigen-specific T-cell clonal expansion but also to understand the functional programs and related phenotypes of the responding and memory Th cells.

Single-cell RNA sequencing (scRNA-Seq) techniques have shed light on the diversity of Th cell programs (17–20), among which classical subsets previously described on the basis of the cytokine release profiles and the surface marker expression patterns have to find their place. The expression of transcripts encoding characteristic surface markers is often low in scRNA-Seq datasets, with indirect correlations between mRNA abundance and protein density (21, 22). This problem can be overcome to some extent through the incorporation of protein-level expression data into single-cell experiments, a task that is essentially implemented in CITE-Seq technology (cellular indexing of transcriptomes and epitopes by sequencing), which makes use of barcoded antibodies directed against markers of interest expressed on the cell surface (23–25).

In this paper, we report on an approach to the mapping and clonal tracking of sorted lymphocyte subsets within scRNA-Seq data, termed TCR-Track. This approach makes use of immune repertoires from sorted lymphocyte subsets, which are then mapped to scRNA-Seq+TCR-Seq datasets obtained from the same donors, employing natural barcodes in the form of sequence-defined T-cell receptors (TCRs). We demonstrate that this method accurately maps phenotypically defined Th subsets within the scRNA-Seq landscape. We further integrate the scRNA-Seq, TCR-Track, and CITE-Seq outputs of 122 donors to provide a comprehensive Th scRNA-Seq reference dataset.

TCR-Track also allowed us to trace the positioning of subset-specific T-cell clones obtained from the deep bulk TCR profiling of the sorted subsets after a 4-year interval. The latter approach, complementing concepts that describe the plasticity of tissue-resident T cells (26, 27), reveals the surprisingly high long-term program stability of the CD4^+^ T-cell clones circulating in human peripheral blood.

In general, based on the integrated reference, we expand upon previous observations of the clonal independence of the circulating Th1, Th2, Th2a, Th17, Th22, and regulatory T-cell (Treg) subsets (28). We also disentangle the interrelations between the subsets classically sorted as Th17 and Th22 and demonstrate a prominent clonal overlap between the Th1 and cytotoxic Th subsets, supporting their common lineage (29, 30).

Finally, we report on an association between severe acute respiratory syndrome coronavirus 2 (SARS-CoV-2) infection and the transient but global type 1 interferon (IFN) activation of naive CD4^+^ T cells, as well as a link between an increased proportion of IFN-induced Th cells and a moderate course of the disease.

## Results

### TCR-Track

**Figure 1:**
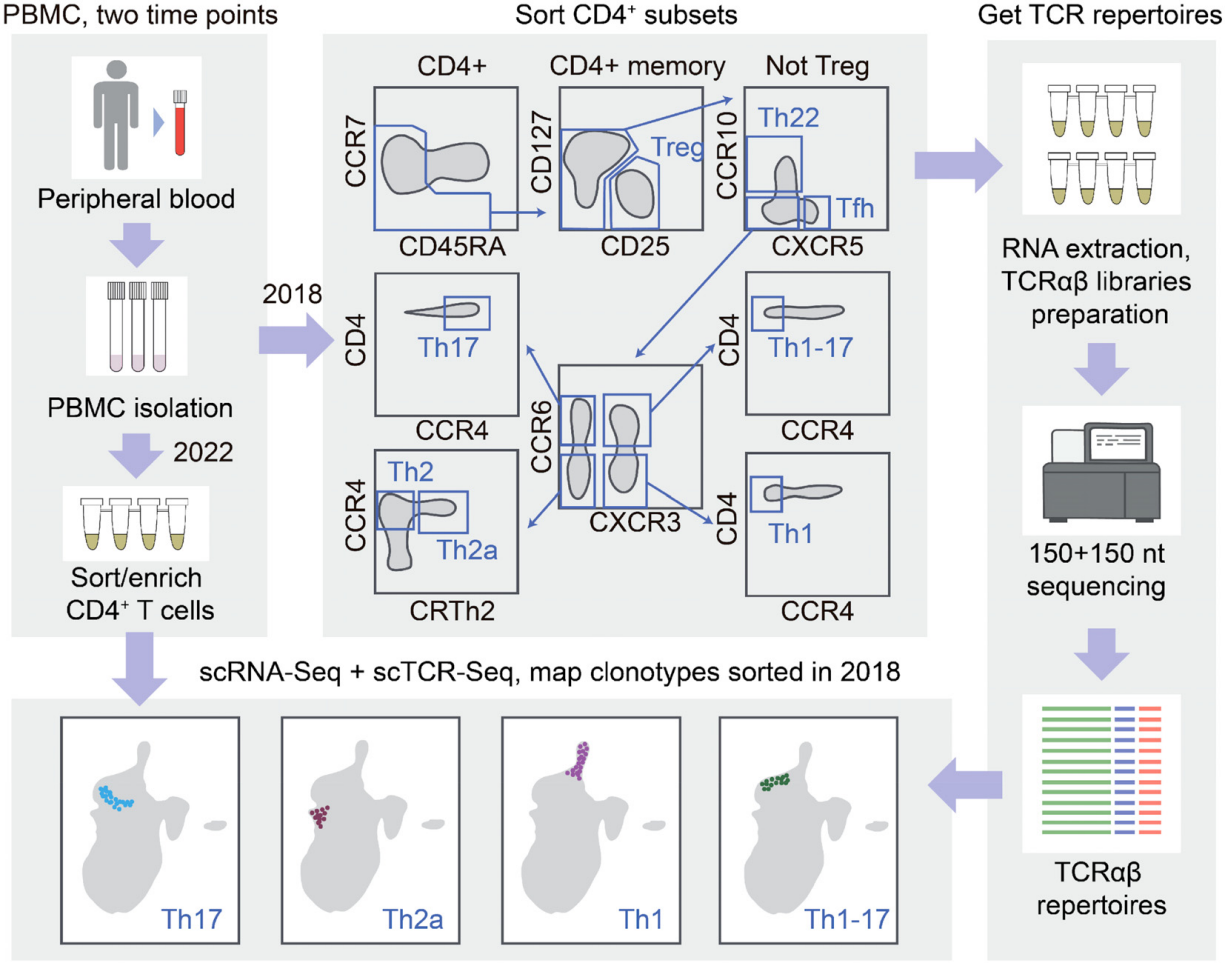
T-cell receptor (TCR)-based mapping of sorted T helper (Th) cell subsets in the single-cell RNA sequencing (scRNA-Seq) data. The Th cell subsets were isolated using fluorescence-activated cell sorting (FACS) with the classic surface markers. RNA-based TCRα and TCRβ repertoires were obtained. After 4 years, scRNA-Seq and scTCR-Seq analysis was performed for the same donor CD4^+^ T cells. Th clones were localized within the scRNA-Seq landscape using TCRs as natural barcodes. Compact positioning of each Th repertoire localized the corresponding T-cell scRNA-Seq clusters and revealed the long-term stability of the circulating Th clonal programs.

Functional subtypes of human Th cells are classically distinguished based on the surface markers, such as CD127, CD25, CCR10, CXCR5, CXCR3, CCR6, CCR4, and CRTh2. In this study, we conceptualized the annotation of Th cells in the scRNA-Seq data powered by overlapping the scTCR-Seq repertoires and the TCR repertoires of Th cell subsets sorted using fluorescence-activated cell sorting (FACS), TCR-Track. We hypothesized that, due to the relatively high phenotypic stability of the Th cell subsets (28), this approach could map the sorted Th subsets within the scRNA-Seq data (**Figure 1**).

**Figure 2:**
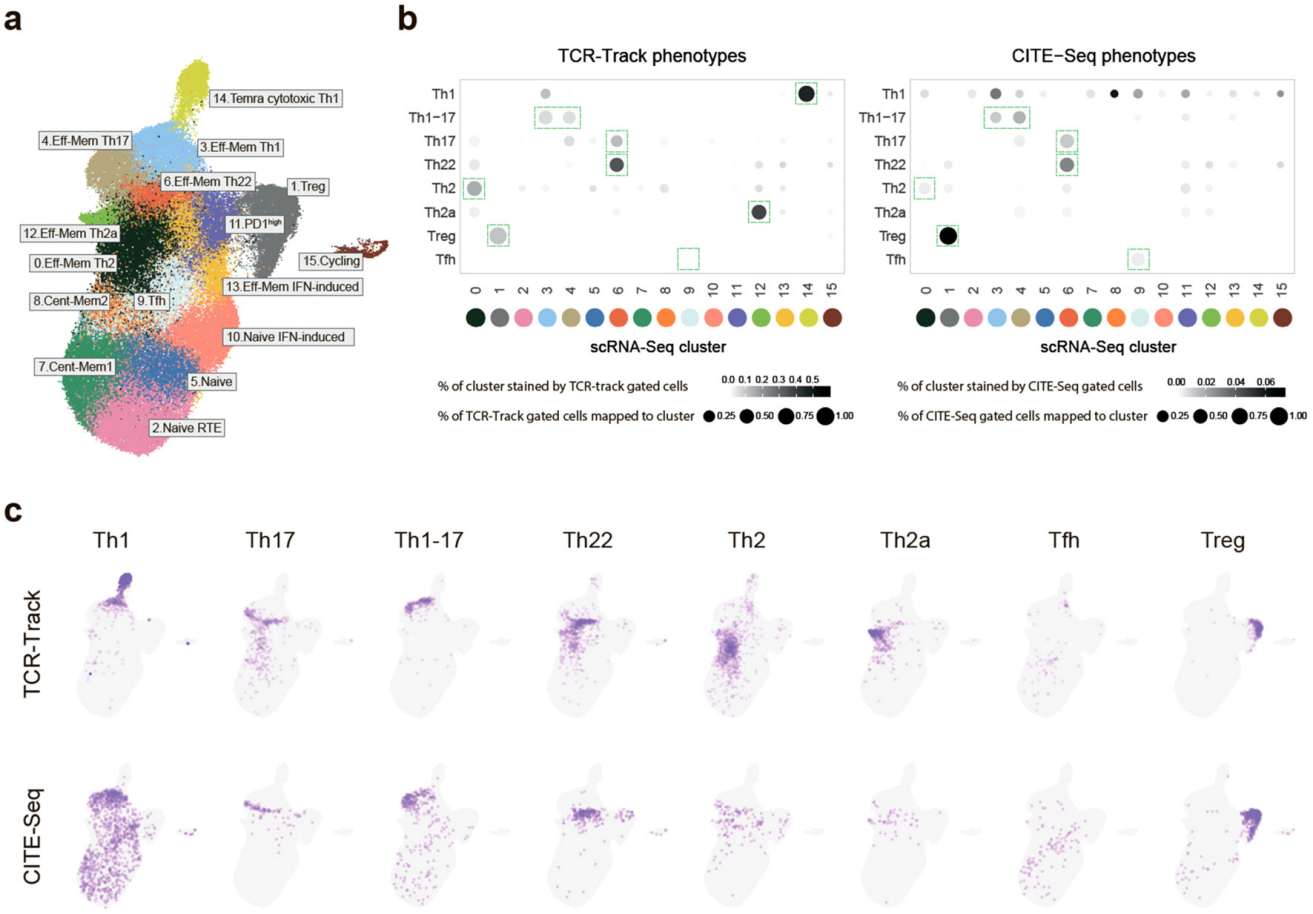
Mapping of the classic T helper (Th) cell subsets with single-cell RNA sequencing (scRNA-Seq). **(a)** Uniform manifold approximation and projection (UMAP) visualization of the reference scRNA-Seq dataset of peripheral blood Th cells. Dataset built via Seurat integration of publicly available data and our scRNA-Seq data. The proposed classification is based on previous knowledge and the findings of the current work. **(b)** Dot plots summarizing the positioning of the sorted (TCR-Track) and *in silico* gated (CITE-Seq) Th subsets within the scRNA-Seq clusters shown in **(c)**. For normalization, 20,000 randomly selected scRNA-Seq cells with matched CITE-Seq or TCR-Track data were used for each plot. *Dot intensity* indicates the stained proportion of the scRNA-Seq cluster. *Dot size* denotes the proportion of the TCR-Track-identified or the *in silico* CITE-Seq-based gated scRNA-Seq cells mapped to the scRNA-Seq cluster. *Green dashed rectangles* indicate the dominating scRNA-Seq cluster. **(c)** UMAP plots showing the localization of the TCR-Track and CITE-Seq defined subsets. TCRβ clonotypes were used to define the Th subsets in the TCR-Track method. Expression of the surface markers was used to gate the Th subsets in the CITE-Seq-based annotation. The *color intensity* in TCR-Track is proportional to the clonal frequencies in the original sorted Th bulk TCRβ repertoires.

We exploited the previously obtained TCRα and TCRβ repertoires of the Th1, Th17, Th1-17, Th22, Th2, Th2a, Treg, and T follicular helper (Tfh) CD4^+^ T-cell subsets sorted rigorously from the peripheral blood of healthy donors (28). Three participants of the latter study were available for repeated blood donation at the time of the current study. We performed paired scRNA-Seq and scTCR-Seq profiling from their sorted effector/memory CD4^+^ T cells (gated as CD4^+^, NOT CCR7^+^CD45RA^+^ cells to deplete naive T cells). The obtained scRNA-Seq data were then integrated with the CD4^+^ scRNA-Seq reported in (31) in order to: 1) increase the power of the downstream analysis such as clustering and uniform manifold approximation and projection (UMAP) visualization on a larger number of donors and 2) enable comparison/complementation of the mapping with the CITE-Seq method employed in the latter work. This resulted in a reference dataset composed of 147,677 cells (**Figure 2a**), without notable donor-specific or study-specific batch effects (**Supplementary Figures S1**-**S3**).

**Figure 3:**
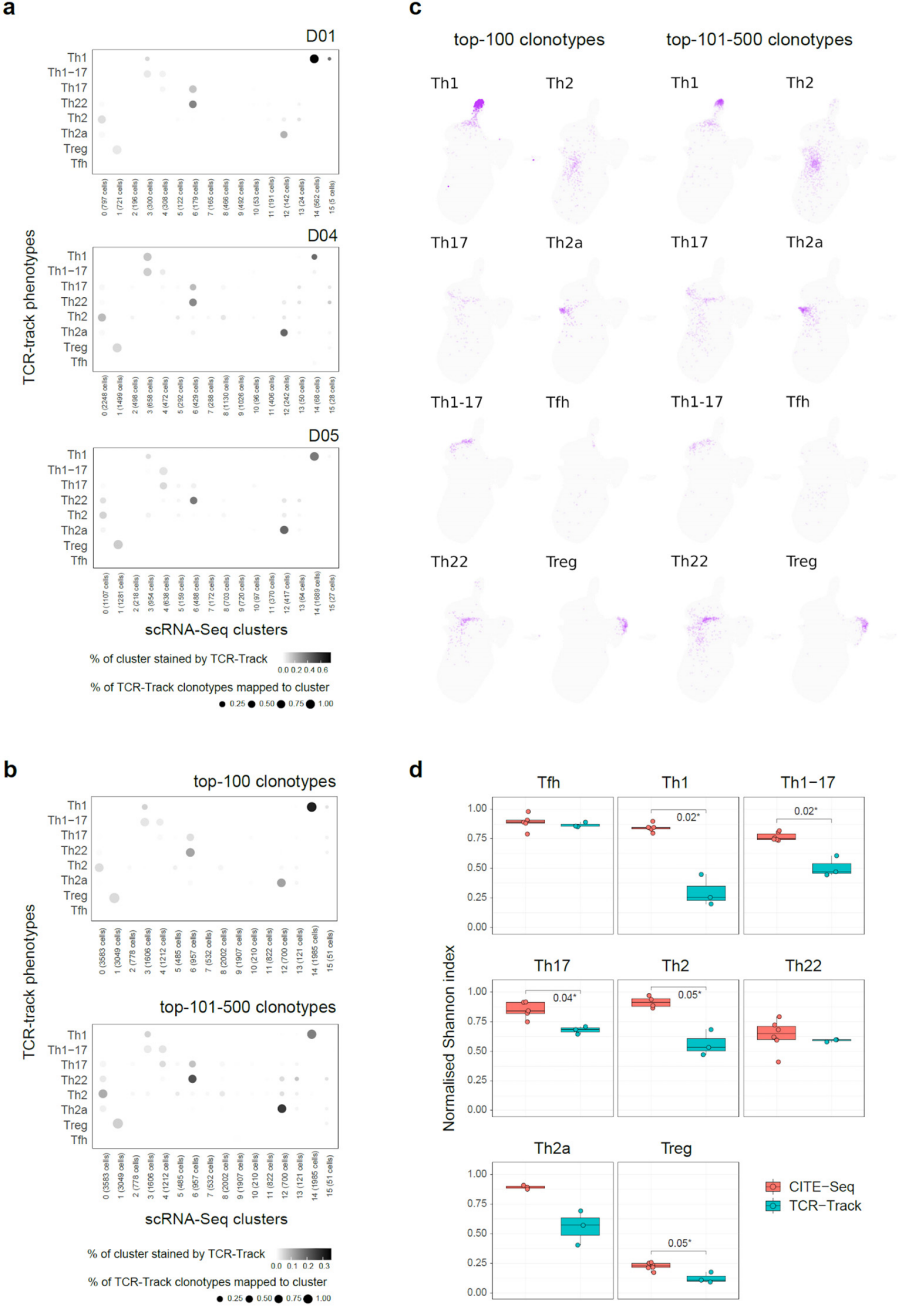
TCR-Track reproducibility and clonality dependence. **(a)** Dot plots summarizing the clonal positioning of the sorted T helper (Th) cell subsets (TCR-Track) within the single-cell RNA sequencing (scRNA-Seq) clusters, shown separately for each donor. **(b)** Dot plots summarizing the clonal positioning of the top 100 and the top 101–500 largest clonotypes from the sorted Th subsets (TCR-Track) within the scRNA-Seq clusters. **(c)** Uniform manifold approximation and projection (UMAP) plots showing the clonal positioning of the top 100 and the top 101–500 largest clonotypes from the sorted Th subsets (TCR-Track) within the scRNA-Seq clusters. TCRβ clonotypes were used to define the Th subsets in the TCR-Track method. The *color intensity* in TCR-Track is proportional to the clonal frequencies in the original sorted Th bulk TCRβ repertoires. **(d)** Relative accuracy of subset mapping to the specific scRNA-Seq clusters measured as normalized Shannon index, which reflects the unevenness of the cell distribution across clusters (Wilcoxon rank-sum test; see *Methods*). The lower the value, the more focused is the mapping.

In order to link the scRNA-Seq clusters to the classic Th phenotypes, the TCRβ clonotypes of the sorted Th subsets were mapped using single-cell T-cell receptors (scTCRs) as natural barcodes. Remarkably, the T-cell clones from each of the sorted Th subpopulations formed clearly defined spots on the scRNA-Seq UMAP (**Figures 2b, c**). Nearly identical results were obtained for the TCRα repertoire mapping (**Supplementary Figure S1B**). The mapping was also reproducible across the three donors (**Figure 3a**; **Supplementary Figure S1C**).

This clear positioning of the Th subset clones allowed us to exploit TCR-Track to build “correspondence between the nomenclatures” of classic immunology (based on the surface molecules and flow cytometry; these are the subsets that can be physically sorted and investigated *in vitro*) and the scRNA-Seq landscape (based on gene expression at the RNA level), filling the gaps of potential miscorrelation between the surface proteins, the FACS sorting sensitivity, and the mRNA expression levels, as discussed in more detail below.

**Figure 4:**
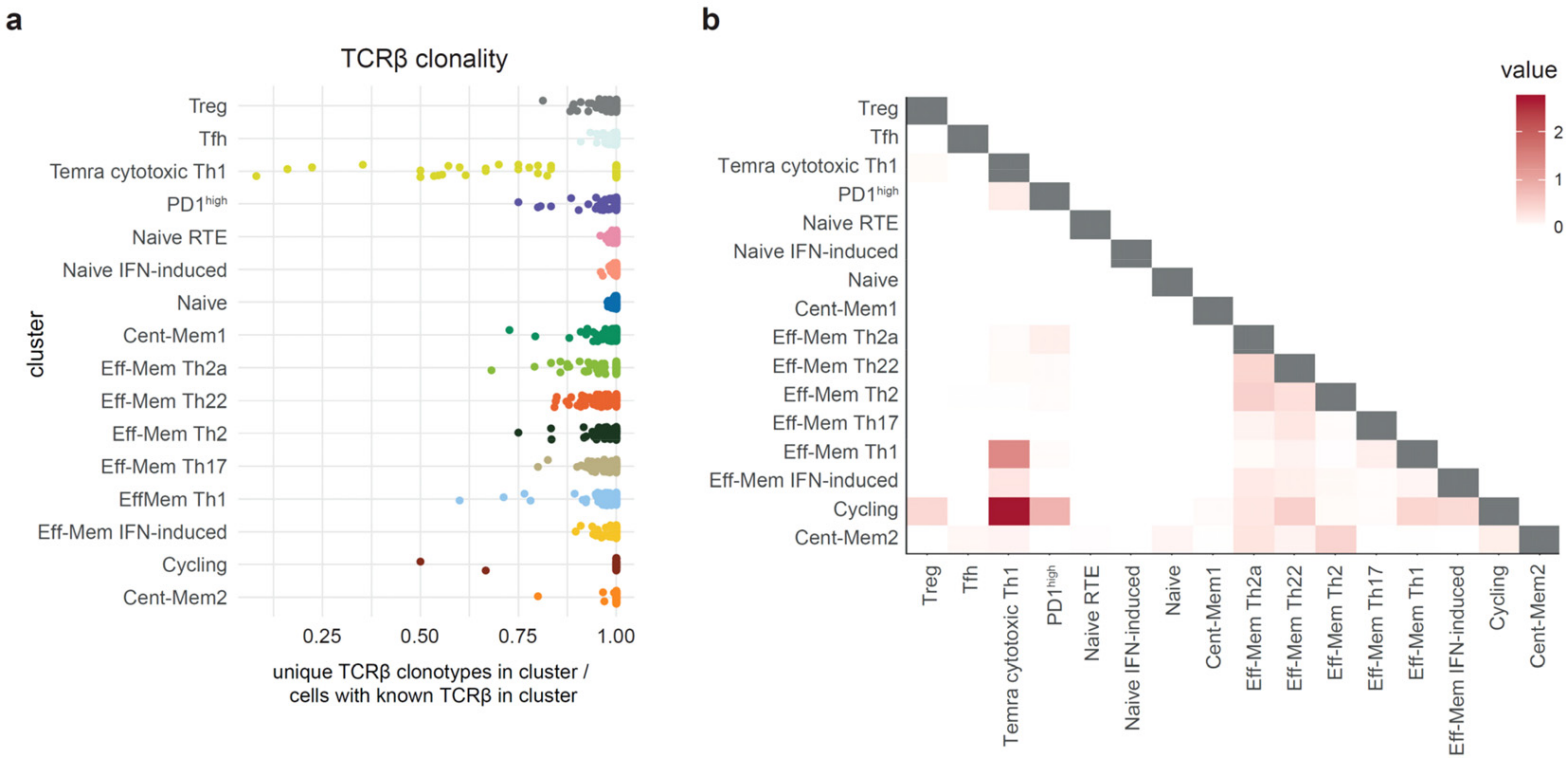
Clonality and clonal overlap between the T helper (Th) cell clusters. **(a)**. Relative clonality of the single-cell RNA sequencing (scRNA-Seq) Th clusters represented as unique TCRβ CDR3/cell count ratio. *Each dot* represents one donor. **(b)** Heatmap visualization of the clonal (nucleotide-defined TCRβ CDR3) overlaps between the scRNA-Seq clusters measured as the number of shared clonotypes between the clusters of the same donor (122 donors used) divided by the number of clonotypes in each cluster (D metrics in VDJtools). The D metric is multiplied by a scale factor (10^6^) and then log2(1 + *x*) transformed for comprehensive values (32, 33).

Mapping of the top 100 and the top 101–500 largest clonotypes from each Th subset produced similar results, indicating that there is no prominent dependence of the TCR-Track performance on clonality (**Figures 3b, c**). Most of the scRNA-Seq clusters showed low clonality (32, 33) in the scTCR-Seq data, thus excluding strong clonal-driven biases in the TCR-Track-based annotation (**Figure 4a**).

In addition, we annotated the same surface marker-defined Th subsets within scRNA-Seq using CITE-Seq. To this end, we used the CITE-Seq data from (31) and performed sequential *in silico* sorting-like gating, as shown in **Supplementary Figure S4**. The CITE-Seq-based mapping was generally consistent with the TCR-Track data analysis, but with the latter resulting in much more accurate cluster annotations (**Figures 2b, c**; **Supplementary Figures S1B–D**, **S5**, **S6**). The higher accuracy of the TCR-Track approach was statistically confirmed (**Figure 3d**). This suggests that TCR-Track represents a more reliable method for the mapping of T-cell subsets, with many additional options provided by the opportunity to track T-cell clones in time and space and across different methods, employing TCR as a natural barcode.

**Figure 5:**
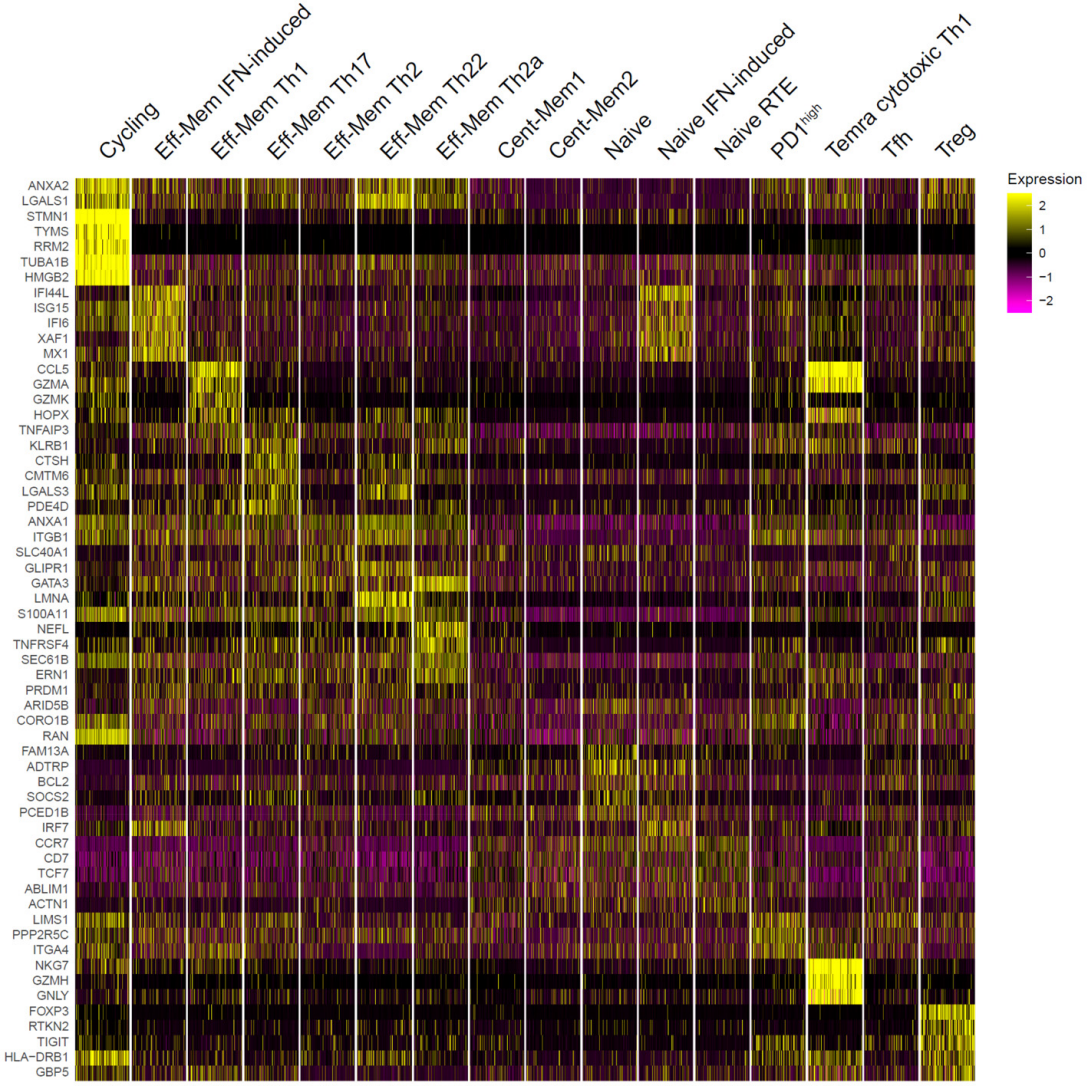
Top genes that distinguished peripheral T helper (Th) single-cell RNA sequencing (scRNA-Seq) clusters. The top 5 differentially expressed genes per cluster are shown. For visualization, 500 cells were randomly selected from each cluster.

Based on the integrated data of the scRNA-Seq, TCR-Track, and CITE-Seq outputs of the 122 donors, taking into account the findings and considerations of Yasumizu et al. (18), a comprehensive peripheral blood Th scRNA-Seq reference map with 16 major Th clusters was proposed (**Figures 2a**, **5**, **Supplementary Table S1**, **Supplementary Figure S6**).

### Correspondence between classic Th subsets and scRNA-Seq clusters

TCR-Track positioned the TCRβ clonotypes of the sorted Treg (CD25^high^CD127^low^), Th22 (CXCR5^−^CCR10^+^), Th2 (CCR10^−^CXCR5^−^CCR6^−^CXCR3^−^CCR4^+^CRTh2^−^), and Th2a (CCR10^−^CXCR5^−^CCR6^−^CXCR3^−^CCR4^+^CRTh2^+^) cells to the corresponding unique scRNA-Seq clusters, clearly fixing their localization in the peripheral blood scRNA-Seq landscape (**Figure 2**).

The sorted Th1 (CCR10^−^CXCR5^−^CCR6^−^CXCR3^+^CCR4^−^) cells mapped well to both the Th1 and Temra (effector memory T cells re-expressing CD45RA) cytotoxic clusters (34–36) (**Figure 2**), coinciding with the plasticity/lineage origin observations, as discussed below.

The sorted Th17 (CCR10^−^CXCR5^−^CCR6^+^CXCR3^−^CCR4^+^) cells mapped to both the Th17 and Th22 clusters, creating uncertainty that is successfully disentangled below.

The sorted Th1–17 (CCR10^−^CXCR5^−^CCR6^+^CXCR3^+^CCR4^−^) cells mapped to tight and linked zones within the Th17 and Th1 clusters (**Figure 2**; **Supplementary Figures S1B, C**). Of note is that these zones do not coincide with any of the scRNA-Seq clusters obtained at any UMAP resolution (**Supplementary Figure S7**), which warrants a more in-depth, focused investigation.

The sorted Tfh (CCR10^−^CXCR5^+^) subset was found in two dissimilar scRNA-Seq clusters (designated as Tfh and Th1) that differed in the expression of CXCR5 and CXCR3. This points to the functional heterogeneity of the Tfh subset (37) and the potential relations between the Tfh and Th1 subsets (17), which warrant a more in-depth investigation.

The “Tnaive SOX4” cluster of Sakaguchi et al. (18) corresponds to the cluster we designated as “Naive RTE” (recent thymic emigrants) based on the expression of CD31 (PECAM1) (38, 39) and on the expression label transfer from the umbilical cord blood CD4^+^ T-cell scRNA-Seq (40). The “Tnaive act” cluster of Yasumizu et al. (18) corresponds to the cluster we designated as “Naive” based on the expression of BCL-2 involved in the homeostatic proliferation of naive T cells (41). The “Tnaive” cluster of Yasumizu et al. (18) corresponds to the cluster we designated as “Central memory 1” based on the observed clonality (**Figure 4a**).

We also distinguished the “PD1^high^” cluster based on the expression of *PDCD1* and the “Cycling” cluster based on the expression of *MKI67* and other cell proliferation markers (**Figure 5**; **Supplementary Figure S5A**).

### Naive and effector IFN-induced clusters

**Figure 6:**
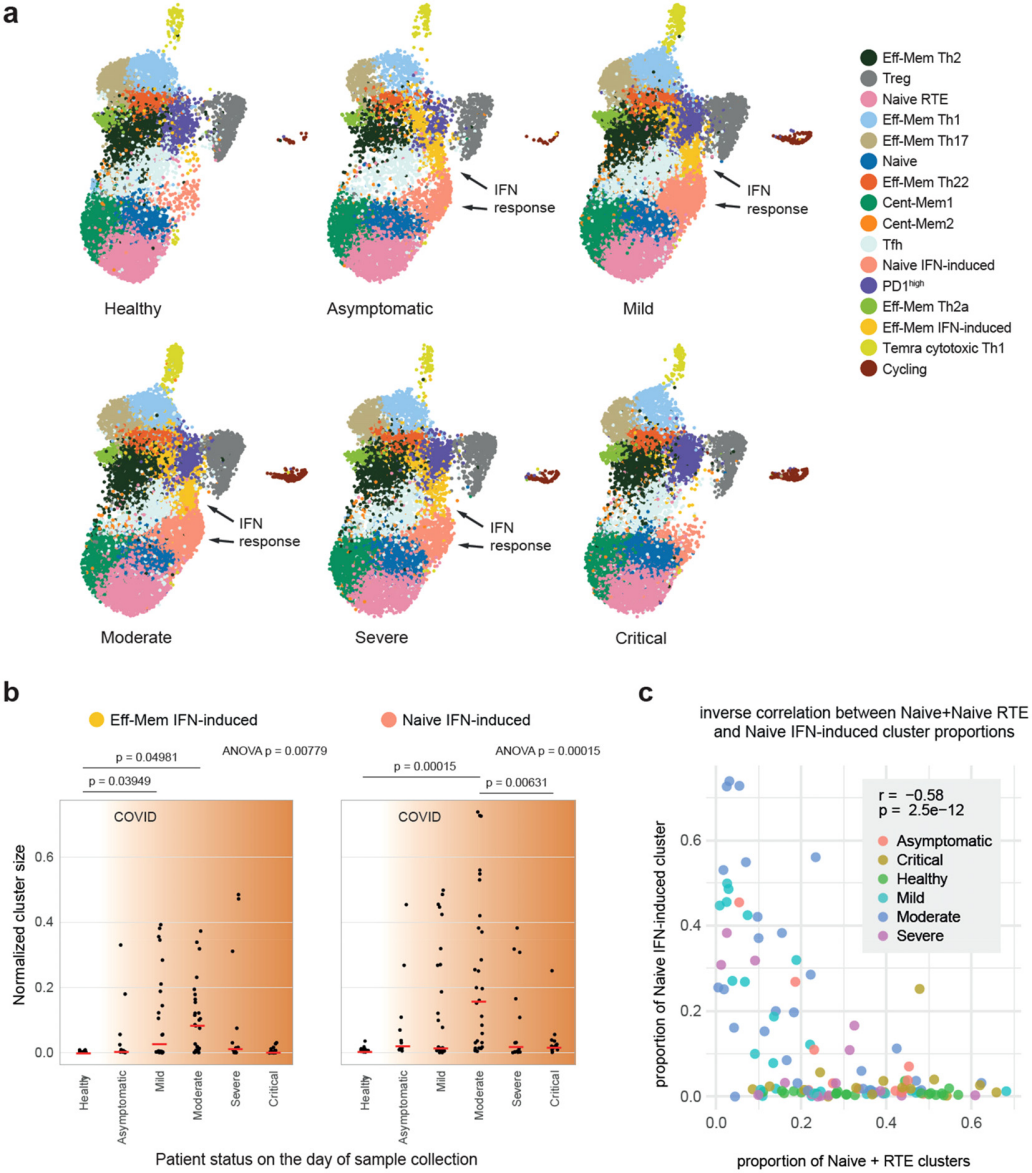
Interferon (IFN) response clusters in healthy donors and coronavirus disease 2019 (COVID-19) patients. **(a)** Uniform manifold approximation and projection (UMAP) plots grouped by disease severity. The “Eff-Mem IFN response” and “Naive IFN response” clusters are nearly absent in healthy individuals. **(b)** Proportion of the samples occupied by the “Eff-Mem IFN response” or the “Naive IFN response” cluster (normalized cluster size). Medians are shown as *red lines*. The *p*-values for ANOVA are shown on *top*. *Post-hoc* pairwise analysis was performed with Tukey’s honest significant difference (HSD) test, and *p* < 0.05 are shown. **(c)** Inverse correlation between the proportions of Naive+Naive RTE *versus* Naive IFN-induced subsets within T helper (Th) cells. *Color* indicates the patient status on the day of sample collection. *r* is Pearson’s correlation coefficient.

Two of the peripheral Th scRNA-Seq clusters, designated as “Naive IFN-induced” and “Effector-memory IFN-induced,” are clearly associated with type 1 IFN response. The effector memory IFN-induced cluster, described earlier (42, 43), was nearly absent in the blood from healthy donors, but was well detectable in patients with coronavirus disease 2019 (COVID-19) (**Figures 6a, b**), probably representing the typical behavior of Th cells in acute viral infection (43). Notably, this cluster was almost undetectable in most critical COVID-19 patients, which emphasizes the importance of IFN response in acute viral infection (44). The “Naive IFN-induced” cluster demonstrated a similar behavior and was inversely correlated with the proportion of “Naive RTE” and “Naive” clusters, suggesting systemic and transient IFN-induced activation of conventional naive T cells in acute viral infection (**Figures 6a–c**; **Supplementary Figure S2**). A similar observation of the “Naive IFN-induced” cluster has been recently reported in COVID-19 and systemic lupus erythematosus data (45).

The “Effector-memory IFN-induced” cluster, predominantly observed in patients with COVID-19, likely represents the activation of effector memory Th cells in response to IFN signaling. This response is crucial for controlling viral replication and coordinating the early antiviral immune response. The near absence of this cluster in critical COVID-19 cases suggests a failure or dysregulation of the IFN pathway, which has been previously linked to severe disease progression.

The “Naive IFN-induced” cluster, which was inversely correlated with the conventional naive T-cell populations, reflects the systemic activation of naive T cells triggered by IFN signaling. This activation might provide a foundation for the preferential priming of new clonal expansions with antiviral programs, potentially driving a shift toward a Th1 phenotype. This hypothesis warrants further investigation.

The differential presence of the “Effector-memory IFN-induced” and “Naive IFN-induced” clusters across disease severities underscores their potential as biomarkers for monitoring IFN pathway activity. Early detection of diminished IFN responses could help identify patients at higher risk of severe disease, enabling timely and targeted therapeutic interventions.

### Phenotypic *versus* intrinsic program plasticity of Th cells

To evaluate the relationships and cross-subset plasticity of the T-cell clones, the cluster stability at different clustering resolution levels (**Supplementary Figure S7**) and the clonal intersections between the clusters were analyzed based on the scTCR-Seq data (**Figure 4b**). We previously suggested the notable plasticity between the Th22/Th17, Th17/Th2, and Th2/Th2a subsets based on the corresponding intersections of the sorted Th cell subset repertoires (28). However, the new data on clonal overlaps and cluster stability at various resolutions in the current study prompted us to partially reconsider these interpretations.

Indeed, the T-cell clones sorted as classic Th17 subset (gated as NOT CD25^high^CD127^low^, CCR10^−^CXCR5^−^CCR6^+^CXCR3^−^CCR4^+^) were found in both the Th17 and Th22 scRNA-Seq clusters (**Figure 2b**). However, the clonal overlap between these two clusters was low (**Figure 4b**), while the stability of both clusters was high (**Supplementary Figure S7**). Furthermore, the clones sorted as classic Th22 (gated as NOT CD25^high^CD127^low^, CXCR5^−^CCR10^+^) were almost exclusively found in the Th22 scRNA-Seq cluster, indicating their self-standing nature. The independent origin of the human Th22 clones was initially suggested (46) and reported in mouse models (47–49). Distinct TCR repertoire features also supported the existence of the clonally discrete Th22 subset (28).

We interpreted these observations as follows. The population classically sorted by phenotypic markers and described as Th17 actually represents the mixture of *bona fide* Th17 and Th22 cells (those of the latter that are stained as CCR10-negative). In contrast, the classically sorted CCR10^+^ Th22 subsets mostly coincide with the corresponding scRNA-Seq cluster, representing a relatively pure population of uniformly programmed *bona fide* Th22 T cells (**Table 1**).

**Table 1:** Correspondence between the classically sorted peripheral blood T helper (Th) cell subsets and the stable scRNA-Seq clusters according to TCR-Track.

Classic name	FACS-sorted, gated as CD4^+^, NOT CCR7^+^CD45RA^+^ (effector memory CD4^+^ T cells)	Correspondence to stable scRNA-Seq clusters (*bona fide* Th programs)
Th1	NOT CD25^high^CD127^low^, CCR10^−^CXCR5^−^CCR6^−^CXCR3^+^CCR4^−^	5:1 mixture of the Temra cytotoxic Th1 and Th1 clusters
Th1-17	NOT CD25^high^CD127^low^, CCR10^−^CXCR5^−^CCR6^+^CXCR3^+^CCR4^−^	Independent subset located within linked and tight zones of the Th1 and Th17 scRNA-Seq clusters; requires more in-depth investigation
Th17	NOT CD25^high^CD127^low^, CCR10^−^CXCR5^−^CCR6^+^CXCR3^−^CCR4^+^	3:2:2 mixture of the Th22, Th17, and Th2 clusters
Th22	NOT CD25^high^CD127^low^, CCR10^+^CXCR5^−^	7:2 mixture of the Th22 and Th2 clusters
Th2	NOT CD25^high^CD127^low^, CCR10^−^CXCR5^−^CCR6^−^CXCR3^−^CCR4^+^CRTh2^−^	6:1 mixture of the Th2 and central memory clusters
Th2a	NOT CD25^high^CD127^low^, CCR10^−^CXCR5^−^CCR6^−^CXCR3^−^CCR4^+^CRTh2^+^	3:1 mixture of the Th2a and Th2 clusters
Treg	CD25^high^CD127^low^	Treg localized in the zone of “effector Tregs” according to the classification by Yasumizu et al. (18)
Tfh	NOT CD25^high^CD127^low^, CCR10^−^CXCR5^+^	Tfh, Th1 (presumably Tfh1); requires more in-depth investigation

**Figure 7:**
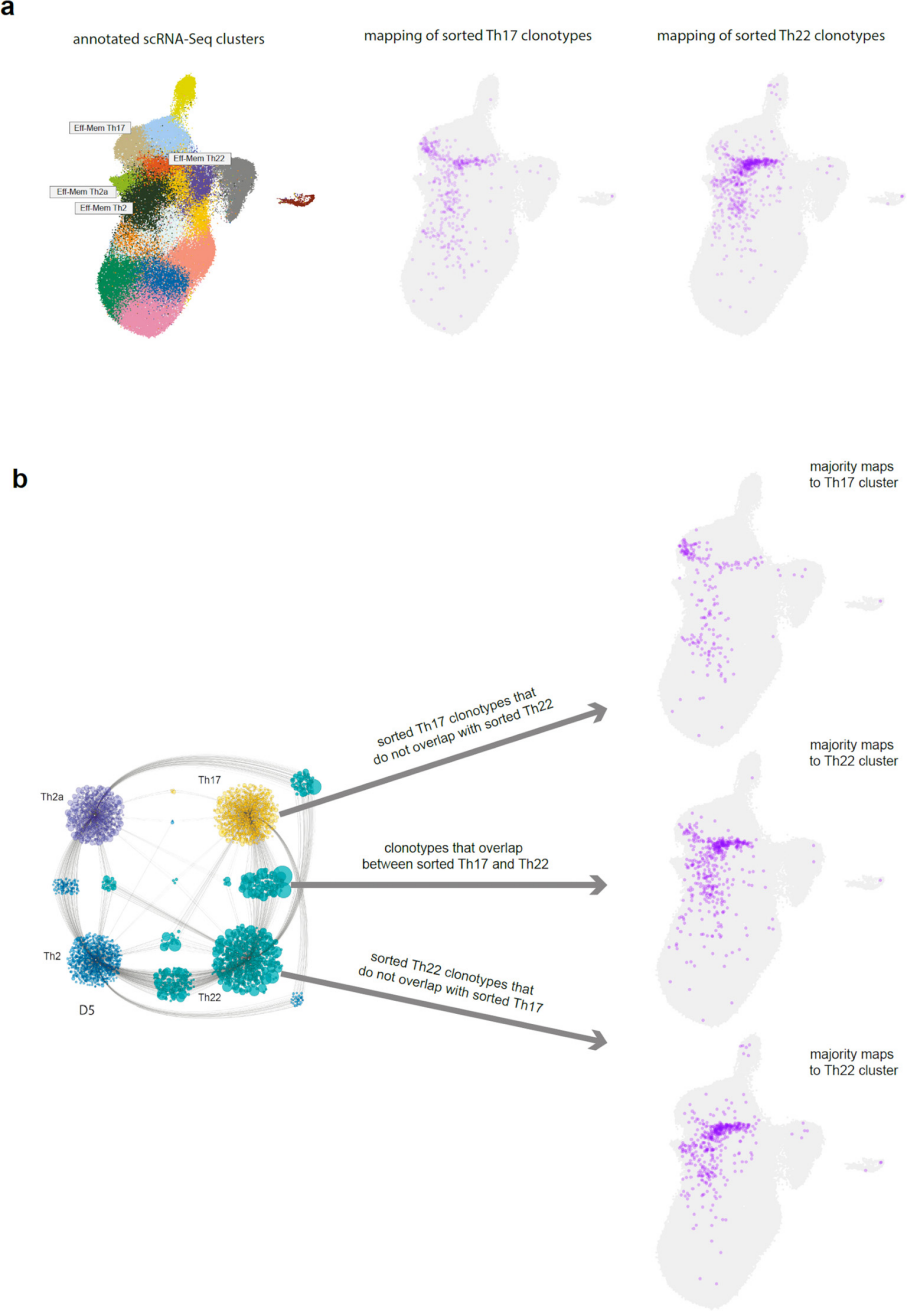
Positioning of the Th17/Th22 shared clonotypes. **(a)** Annotated single-cell RNA sequencing (scRNA-Seq) clusters and TCR-Track positioning of the sorted Th17 and Th22 subsets. **(b)** Uniform manifold approximation and projection (UMAP) positioning of the sorted Th17 clonotypes that do not overlap with the sorted Th22, the sorted Th22 clonotypes that do not overlap with the sorted Th17, and the overlapping clonotypes. Cytoscape network plot adapted from (28).

To confirm this hypothesis, we plotted those TCRβ clonotypes that were found as shared between the sorted Th22 and Th17 subsets in (28), which showed that these clonotypes were almost exclusively localized within the Th22 scRNA-Seq cluster, confirming their *bona fide* Th22 program. In contrast, the sorted Th17 clonotypes that did not overlap with the sorted Th22 predominantly mapped to the Th17 scRNA-Seq cluster, confirming their *bona fide* Th17 program (**Figure 7**).

Altogether, these data clearly support the self-standing nature of the Th17 and Th22 subsets. That being said, some clonal overlap between the Th2, Th2a, and Th22 scRNA-Seq clusters was observed (**Figure 4b**), thus leaving room for the moderate long-term plasticity between these three subpopulations (49).

The Th1 and Temra cytotoxic Th1 clusters demonstrated notable clonal overlap (**Figure 4b**), indicating that the former could convert into the latter, as some of the recent studies suggest (29, 30). Correspondingly, the positioning of the sorted Th1 TCRβ clonotypes to the Th1 and Temra cytotoxic clusters may reflect terminal maturation of the Th1 clones. The highest clonality was also observed for the cluster annotated as the Temra cytotoxic Th1 subset, which is in line with previous reports (35) (**Figure 4a**).

**Table 1** summarizes the observed correspondence between the sorted peripheral blood Th subsets and the scRNA-Seq clusters. In the proposed scRNA-Seq classification shown in **Figure 2a**, we gave priority to the cells partitioning into stable scRNA-Seq clusters (**Supplementary Figure S7**), while the TCR-Track data were used to match the classical surface phenotype-based sorted T-cell subsets with those clusters.

### Programs of peripherally circulating Th clones are stable in time

Of note is that the T-cell repertoires of the sorted subsets were obtained in 2018, while the scRNA-Seq experiment was performed on samples derived in 2022. Despite the 4-year distance, TCR-Track mapped the sorted clones to clearly defined positions, which was generally limited to one or two neighboring scRNA-Seq clusters (**Figure 2**; **Supplementary Figure S1**), indicating the long-term program stability of the CD4^+^ T-cell clones circulating in human peripheral blood.

The current behavior of T-cell programs in tissues, in the context of the ongoing interaction with pathogens, microbiota, and the environment, can be associated with much more plastic behavior, forming a “continuum” where clear separation of the Th programs may be more problematic (26, 27, 50). However, our results suggest that program imprinting may remain stable in most conditions, and when returning to the challenge-free circulation, the “resting” T-cell memory clones return to their major initial imprint.

This concept is supported by the generally low clonal overlap between the scRNA-Seq clusters, with the exception of the Th1 and Temra cytotoxic Th1 clusters, as well as the natural intersection of “Cycling” Th cells with several differentiated Th clusters (**Figure 4b**).

## Discussion

The architecture of T-cell memory essentially determines the entire pattern of our interaction with the antigens of the surrounding world, our microbiota, and our self-antigens (1, 2). This architecture starts formation in the prenatal period (51–55), is actively formed in the first years of life in the contact with pathogens, airborne and food antigens, and microbiota maturation (56, 57), and then continues to be actively shaped by vaccinations and further contacts with infectious and non-infectious challenges and antigens.

Clonal populations of T cells that are instructively primed by professional antigen-presenting cells (1–3) make decisions about which reaction programs to choose to respond to each specific antigen. They remember these programs as memory clones, forming stable response patterns to familiar challenges, patterns of regulation and cross-regulation of the immune responses to friends and foes.

Mistakes made in such decisions cost us dearly: they lead to autoimmune diseases, inefficient elimination of pathogens, chronic inflammation, and cancer and may essentially underlie the entire phenomenon of inflammaging. Presumably for this reason, some mammalian species are likely to avoid forming such a long-term clonal memory (11). In humans, however, the clonal memory of both CD8^+^ and CD4^+^ T lymphocytes can persist for years and decades (58, 59).

In the present work, TCR-Track allowed us to map the Th subset repertoires on the very same T-cell clones of the scRNA-Seq data obtained for the donor 4 years later, which emphasizes the stability of the huge number of clones accumulated over previous years (60). We also showed that these clones were mapped predominantly or even exclusively within their corresponding and independent scRNA-Seq clusters, highlighting the persistence of program decisions once made by each CD4^+^ T-cell clone. That said, both tissue plasticity (26, 27, 50) and the non-excluded opportunity for the multiple programs acquired by the progeny of a naive T cell, which could potentially be revealed with more in-depth analysis (61), remain relevant.

Repertoire-based TCR-Track annotation also turned out to be a powerful aid in matching the classical subsets of lymphocytes sorted by surface markers with the stable scRNA-Seq clusters that more comprehensively describe the diversity of functional lymphocyte programs. This effort structures our understanding of the functional diversity of Th cells, which is critical for further progress in cancer and autoimmunity immunotherapy and vaccine development.

In particular, focus on eliciting/supporting/exploiting appropriate Th cell programs may qualitatively improve the efficiency of immunotherapeutic interventions, e.g., chimeric antigen receptor T cell (CAR-T) and T-cell receptor T cell (TCR-T) approaches, and cancer vaccines. In autoimmunity, where tolerogenic vaccines (62, 63) and targeted immunotherapeutic interventions (64, 65) are gaining momentum, the precise selection and control of the Th response types is also crucial.

CITE-Seq can also be used to phenotype cell populations on a single-cell level based on the surface markers, which has an advantage of simultaneously measuring hundreds of those. Our work shows that TCR-Track may outperform CITE-Seq in terms of resolution capacity. At the same time, we should indicate that this may be attributed to a technical instability in multiplex CITE-Seq, where a successful CITE-Seq experiment may yield results comparable to those of TCR-Track. In addition, some of the CITE-Seq staining antibodies may affect the cell signaling and transcriptomic profiles, introducing bias in the scRNA-Seq landscape (66). In contrast, in the TCR-Track pipeline, cells stained with surface antibodies for sorting and bulk TCR-Seq are obtained and analyzed independently of the scRNA-Seq/scTCR-Seq experiment.

We anticipate that, in future works, TCR-Track can be used to match and classify the following: 1) CD4^+^ T-cell populations in the peripheral blood, lymph nodes, tertiary lymphoid structures at the sites of chronic inflammation and in the tumor environment, and other tissues in health and disease; 2) diverse Tfh cells (37); 3) known and unknown types of invariant and semi-invariant T cells, such as invariant natural killer T (iNKT), natural killer T (NKT), mucosal-associated invariant T (MAIT), and Crohn’s-associated invariant T (CAIT) cells (67–69); 4) gamma delta T cells (70); 5) central memory and stem cell memory T cells that would probably require deeper scRNA-Seq coverage due to their relatively lower clonality; 6) CD8^+^ T-cell subsets (71); 7) and B-cell functional subsets (72). A limitation of our study is that the Th subset repertoires and the scRNA-Seq data were obtained 4 years apart. Supporting our findings with data collected at the same time point and from the same and different tissues would greatly enhance our understanding of T-cell immunity.

The TCR-Track logic that exploits TCR as a natural barcode opens multiple options to track T-cell clones in time and space to disentangle the complex puzzle of the clonal relations between the subsets (as we did here for Th17 and Th22) and between tissues. For example, one could identify the TCR repertoire of sorted Th subsets from peripheral blood and track this functionality in scRNA-Seq data obtained from the tumor-infiltrating T cells of the same patient, informing on the program plasticity of the T-cell clones in the tumor microenvironment.

The TCR-Track approach has certain limitations that must be taken into account. Firstly, similar to CITE-Seq, the method relies solely on surface markers to define the target cell subpopulations. Secondly, the method is applicable only to T and B cells with specific clonal receptors acting as living barcodes. Thirdly, TCR-Track is based on lymphocyte clonality, where there is a need to capture the same clone in the sorting and scRNA-Seq experiments. Therefore, TCR-Track-based annotation of, e.g., central memory T-cell subsets or peripheral blood Tfh cells, which are almost as diverse as naive T cells (28, 37), may require substantially more in-depth scRNA-Seq and bulk TCR profiling. For the naive T-cell subsets, the application of TCR-Track would be probably limited to innate-like, relatively clonal, naive T-cell subpopulations of fetal origin (53).

In summary, our work:

1. Offers TCR-Track as an approach for the classification of phenotypically defined T- and B-lymphocyte subsets, their exact positioning within the scRNA-Seq landscape, and time tracking.
2. Clarifies the correspondence between the well-studied human peripheral blood Th subsets and scRNA-Seq clusters.
3. Proposes a more accurate, clonally informed CD4^+^ T-cell classification within the scRNA-Seq landscape, delineating the positioning of the Th2, Th2a, Th22, and Tfh clusters and refining several other details. An integrated scRNA-Seq reference dataset of peripheral Th lymphocytes is provided.
4. Shows the long-term program stability and low intrinsic plasticity of the Th memory clones circulating in peripheral blood.
5. Shows a high clonal overlap, suggesting that cytotoxic CD4^+^ T cells differentiate from Th1 clones.
6. Shows that Th17 and Th22 represent clonally independent subsets. The sorted Th17 subset represents a mixture of *bona fide* Th17 and CCR10^low^ Th22 cells.
7. Shows that SARS-CoV-2 infection is associated with transient type 1 IFN activation of naive T cells, a phenomenon which warrants a more in-depth investigation.
8. Shows that efficient response to SARS-CoV-2 infection is associated with the appearance of prominent effector IFN-induced Th cells, while critical COVID-19 is associated with a low presence of the IFN-induced subset.

More generally, we hope that this work advances the study of the role of programmed populations of memory T cells to a new level, making it possible to clearly distinguish the functional nature of each immune response and to investigate the plasticity between T-cell subsets. This level of understanding serves as a necessary stepping stone to the rational development of better immunotherapeutic approaches in oncology and autoimmunity, as well as in vaccine development, where the chosen T-cell programs fundamentally determine the type of immune response and are crucial to the outcome.

## Methods

### scRNA-Seq and scTCR-Seq library preparation and sequencing

Fresh peripheral blood mononuclear cells (PBMCs) from D01, D04, and D05 were stained with anti-CCR7–PE-Cy7 (clone 3D12; BD Biosciences, Franklin Lakes, NJ, USA), anti-CD3–APC-Fire750 (clone SK7; BioLegend, San Diego, CA, USA), anti-CD4–PE-Cy5.5 (clone S3.5; Thermo Fisher Scientific, Waltham, MA, USA), anti-CD14–V500 (clone M5E2; BD Biosciences), anti-CD19–V500 (clone HIB19; BD Biosciences), anti-CD45RA–PE-Cy5 (clone HI100; BioLegend), and LIVE/DEAD Fixable Aqua (Thermo Fisher Scientific). Viable effector/memory CD4^+^ T cells gated as CD3^+^CD4^+^CD14^−^CD19^−^ after excluding CCR7^+^CD45RA^+^ events were sorted into two replicates for D01 and without replicates for D04 and D05 using a custom-modified FACSAria II (BD Biosciences) and loaded onto a Chromium Controller (10x Genomics, Pleasanton, CA, USA). The samples were prepared using a Chromium Next GEM Single Cell 5′ Reagent Kit v2 (10x Genomics). The pooled samples were sequenced with a coverage of 100,000 reads per input cell for scRNA-Seq and 25,000 reads per input cell for scTCR-Seq on a NovaSeq 6000 System with an S4 Flow Cell (Illumina, San Diego, CA, USA).

### scRNA-Seq, scTCR-Seq, and CITE-Seq data analysis

Raw scRNA-Seq and scTCR-Seq fastq files were processed using the *count* and *vdj* pipelines in cellranger (v6.1.2). For scTCRs, only productive in-frame CDR3^−^ and V/J-spanning contigs without the stop codons in the V–J region were selected, and the most abundant TCR chain was used for cells with two relevant transcripts (TRA or TRB). Filtered gene expression matrices were uploaded into the Seurat R package (v4.2.0) (73). The cells containing >10,000 unique molecular identifiers (UMIs) and >10% mitochondrial reads were removed. The data from each donor were paired with the corresponding scTCR data, log-normalized with the *NormalizeData* function, and clustered using the Louvain algorithm. Publicly available processed multimodal scRNA-Seq/scTCR-Seq/CITE-Seq data from PBMCs were downloaded from the ArrayExpress database under accession number E-MTAB-10026. These data were converted into Seurat object, applying the same filtering criteria as those used for the newly generated data. CD4^+^ T-cell clusters were selected based on the average cluster expression of *CD4* and *CD3E* and the cluster annotations provided by the authors. The public dataset was then split by sample origin, log-normalized, and clustered with the Louvain algorithm based on sets of highly variable genes calculated individually for each batch using Seurat functions. The CITE-Seq data, provided as the antibody-derived tag (ADT) counts for 192 features in the processed data, were normalized separately for each batch using the centered log ratio (CLR) method as implemented in Seurat and were used for further analysis. In addition to the filtering steps originally performed by the authors, at this stage, outlier clusters with both low UMI counts and high percentage of mitochondrial genes were removed, as well as clusters expressing *CD8A* and *CD8B* from all individual datasets. Differential expression was calculated using the *FindAllMarkers* function individually on each dataset. Clusters that either highly expressed or contained B-cell, dendritic cell, macrophage, and other non-T-cell markers in the differential expression as per single-cell RNA section of the Human Protein Atlas (https://www.proteinatlas.org/humanproteome/tissue) were removed from the analysis. Clusters that expressed the markers of natural killer (NK) cells without the expression of CD3/TCR were also removed. The stress score was calculated for each cell using the *AddModuleScore* function in Seurat. This included the genes upregulated as a consequence of the dissociation procedure (*BTG1*, *BTG2*, *DDX5*, *DNAJA1*, *DUSP1*, *EEF1A1*, *HSPA8*, *JUN*, *JUNB*, *JUND*, *KAP*, *KLF6*, and *PNRC1*) (74).

The paired scRNA/scTCR-Seq dataset generated in this study contained 23,257 cell barcodes, of which approximately 90% contained the scTCR-Seq data in each of the three donors. The total cell counts for the donors were 4,723, 9,430, and 9,104. For public data, out of the 124,420 cell barcodes from the 119 donors selected for the analysis, approximately 80% contained scTCR-Seq data. In public data, 20% of the cells came from 21 healthy donors, 77% came from 93 donors with COVID-19 infection across several disease severity groups—asymptomatic (9,400 cells), mild (29,807 cells), moderate (24,774 cells), severe (14,303 cells), and critical (17,031 cells)—and 3% came from five donors with non-COVID-19 infection.

Integration of the scRNA-Seq data was carried out using the Seurat reference-based reciprocal PCA protocol with default parameters, with the largest 3′ (Newcastle public data) and 5′ (D05) datasets chosen as references to account for differences in the methodology (73). The percentage of mitochondrial genes and the stress score were regressed out of the integrated dataset as implemented in the *ScaleData* function. The number of dimensions used for running the UMAP and the Louvain clustering algorithm was 25 based on the *ElbowPlot* Seurat function. All of the TCR and immunoglobulin (IG) genes were removed from the variable features used in the principal component analysis (PCA) and from the anchor features used for integration. To identify cluster marker genes, the *FindMarkers* and *FindAllMarkers* functions were used on the scRNA-Seq data slot.

For the label transfer from the UCB data, we used processed T-cell scRNA-Seq data from figshare (https://figshare.com/projects/Single-cell_mapping_of_progressive_fetal-to-adult_transition_in_human_naive_T_cells/76143) and gated CD4 T-cell clusters from umbilical cord blood as described previously. We projected the cluster identities of the integrated data onto this dataset via the Seurat data transfer method (*FindTransferAnchors* and *TransferData* functions). The distribution of the predicted IDs with a confidence score >0.5 was used to support the annotation of cluster 2 as “Naive RTE” (data not shown).

*In silico* CITE-Seq gating was performed sequentially, following the scheme in **Supplementary Figure S4**, and visualized using the R packages “ggplot2” (v3.4.4) and “ggpointdensity” (v0.1.0). The CCR10 marker expression was assessed using scRNA-Seq as CCR10 was not included in the CITE-Seq panel. To quantitatively evaluate the accuracy of the TCR-Track and CITE-Seq cluster mapping, the normalized Shannon–Wiener index, $S_{j}=\frac{\sum_{i=1}^{16}\;p_{j}*\text{log}\left(p_{j}\right)}{\text{log}\left(N\right)}$ , was calculated, where *j* is the gated subset, as in **Supplementary Figure S4** (e.g., Tfh, Th1, or Th1–17); *i* is the scRNA-Seq cluster (e.g., 0, 1, or 2); *p_j_* is the proportion of the gated subset *j* in cluster *i*; and *N* is the total number of cluster *i* with non-zero hits for the gated subset *j*. This measures the evenness of the cell distribution across clusters for each gated subset. The gates for CITE-Seq gating were chosen to minimize the resulting normalized Shannon–Wiener index for each gated subset. For evaluation of the results, the index was separately calculated within three donors in the TCR-Track data and within the five disease severity groups in the public CITE-Seq data (the donors were grouped to make the number of cells comparable in each of the analyses). Only the gated subsets with more than seven cells were included in the analysis.

### Mapping of the FACS-sorted Th subset clonotypes

From each bulk TCRβ repertoire (Th1, Th2, Th17, Th1–17, Tregs, Th2a, Tfh, and Th22) from Kasatskaya et al. (28), the top 500 nucleotide clonotypes were selected to make the analysis uniform and also to exclude even minor cross-contaminations that could happen during cell sorting or sequencing. In cases when replicates were available for the bulk TCR repertoires, only one of the pairs was used in the analysis. Subsequently, for each single cell from D01, D04, and D05, the respective TCRβ clonotype frequencies from the top 500 bulk TCR-Seq Th datasets were added to the Seurat object metadata. These frequencies were used as the transparency encoding variables (aesthetic “alpha” in ggplot2) in **Figures 2c**, **3c** and in **Supplementary Figure S1B** to emphasize large clonotypes. In **Supplementary Figures S1C, D** and **Figure 7**, all of the shown cells have identical transparency values.

### Identification of clonotypes overlapping between the sorted Th17 and Th22 subset repertoires

The overlapping TCRβ nucleotide clonotypes were identified between the sets of the top 2,000 TCR clonotypes from the sorted Th17 and Th22 cell subsets (28). Subsequently, a standard deviation was calculated for the frequencies of each overlapping clonotype in the Th17 and Th22 subsets from the same donor. We selected only those overlapping clonotypes for which this standard deviation was lower than 0.001. In this way, from the list of overlapping clonotypes, we excluded those that were highly abundant in the Th17 subset and were low abundant, but still present, in the Th22 subset, or *vice versa*.

## Data Availability

The datasets presented in this study can be found in online repositories. The names of the repository/repositories and accession number(s) can be found below: https://www.ncbi.nlm.nih.gov/, PRJNA995237. A processed Seurat object with the Th reference (“full_reference_return_model.rds”) is available for download at https://figshare.com/articles/dataset/full_reference_return_model_rds/23790393?file=45860319. Code for reproducing the figures and user guide for mapping user datasets on reference scRNA-Seq dataset of helper T cells with Seurat is available at https://github.com/kriukovav/Thelpers.
