# Supplementary material for: Repertoire-based mapping and time-tracking of T helper cell subsets in scRNA-Seq
Source: Front Immunol. 2025 Apr 4;16:1536302. doi: 10.3389/fimmu.2025.1536302 (PMC12006041; doi:10.3389/fimmu.2025.1536302)
Supplement: Supplementary file 1 [file DataSheet1.pdf]

Supplementary Figures

for

Repertoire-based mapping and time tracking of T helper cell subsets in scRNA-Seq

by

Daniil K. Lukyanov, Valeriia V. Kriukova,
Kristin Ladell, et al.

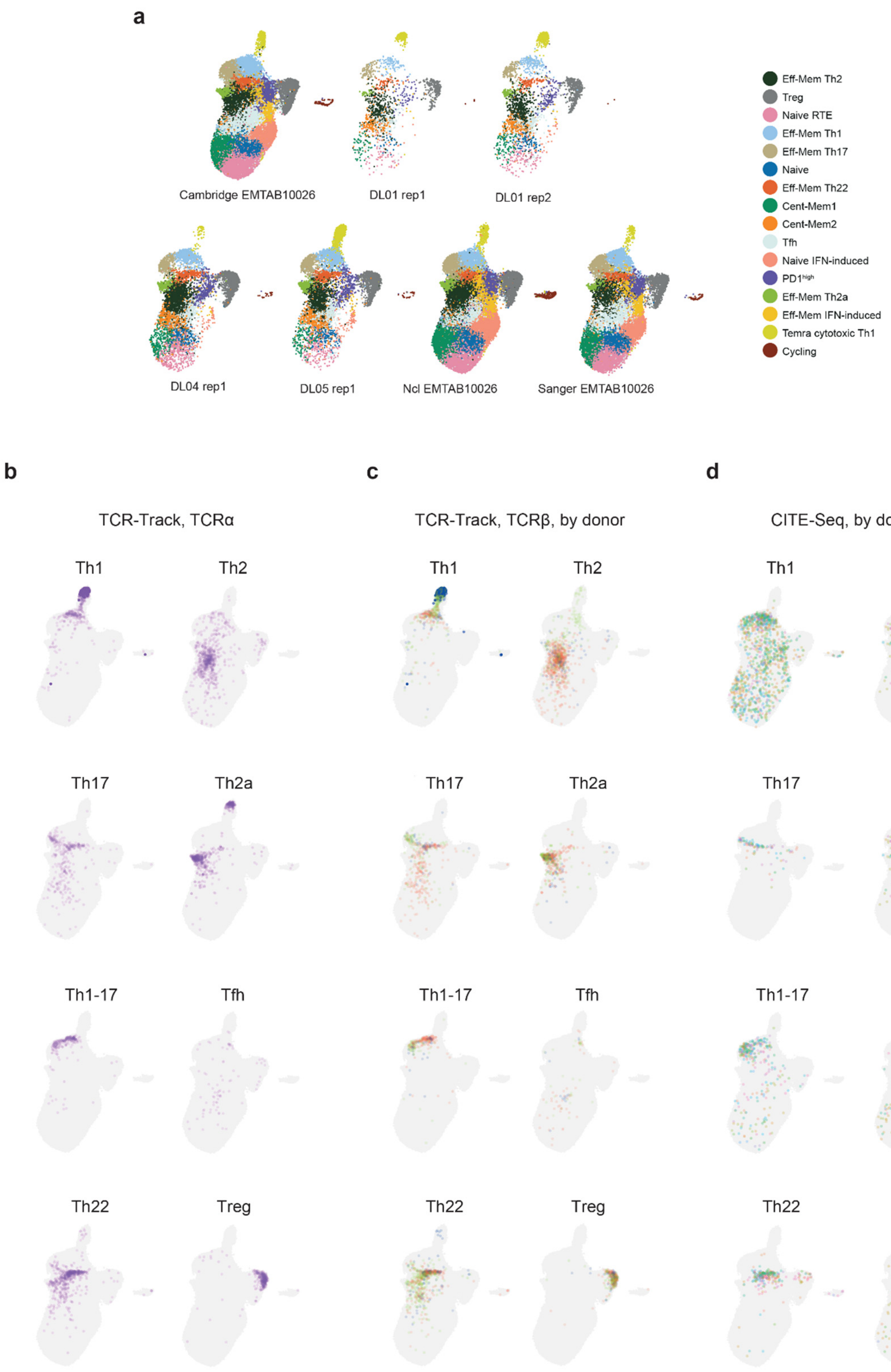

Supplementary Figure 1. Building and characterization of the reference dataset. a. UMAP plots of integrated dataset split by the origin of samples, colored by the assigned cluster. **b**. UMAP plots showing the localization of Sort-Seq-defined TCRα clonotypes. **c**. UMAP plots showing the localization of Sort-Seq-defined TCRβ clonotypes, colored by donor (n = 3). **d**. UMAP plots showing the localization of CITE-Seq-defined cells, gated *in silico* as shown on **Supplementary Fig. 5,** colored by donor (n = 119).

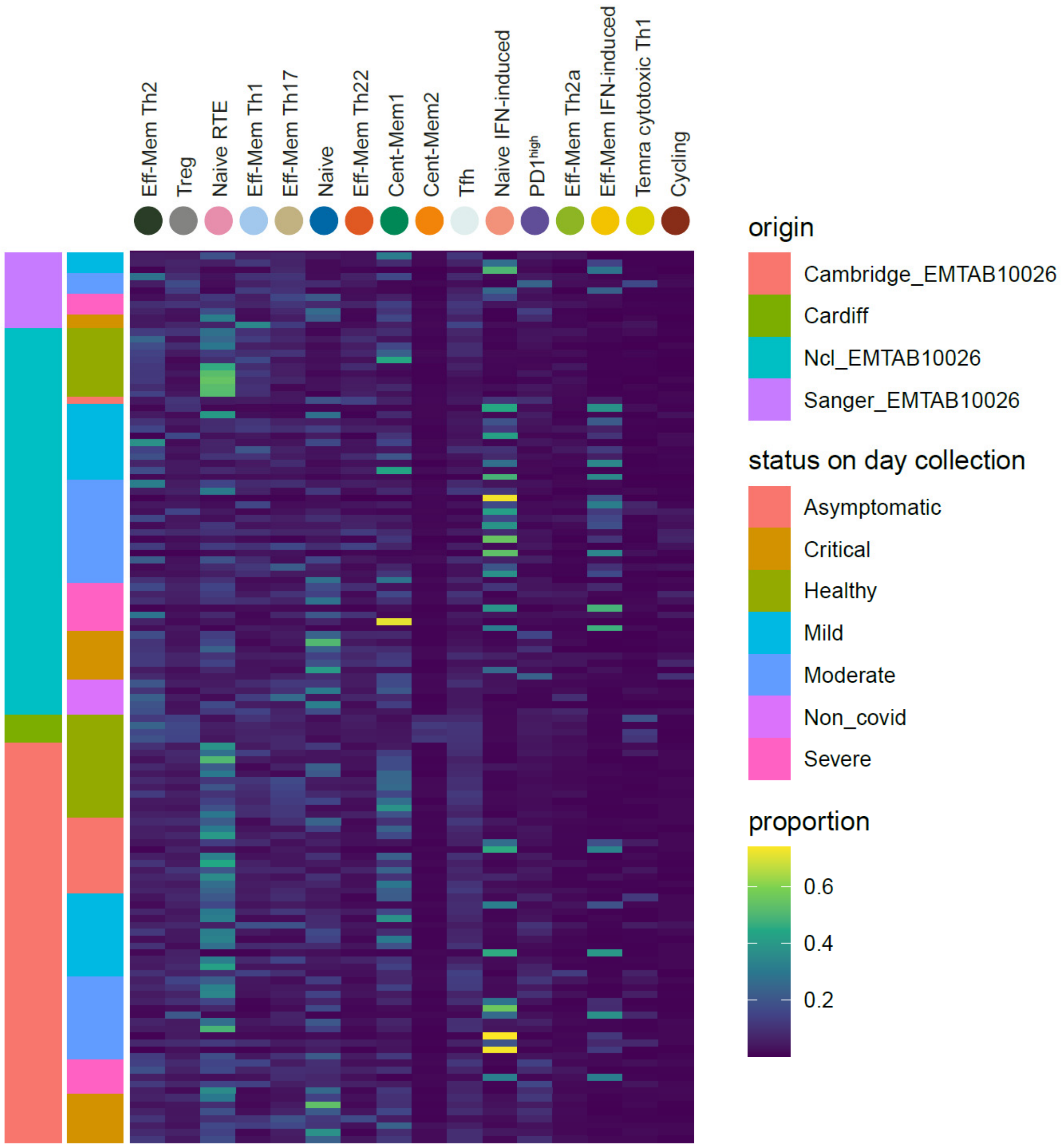

Supplementary Figure 2. scRNA-Seq clusters distribution across studies and COVID disease status. Heatmap shows distribution of scRNA-Seq Th clusters in individual patients. Patients are grouped according to the study and COVID status, as shown on the top with color code.

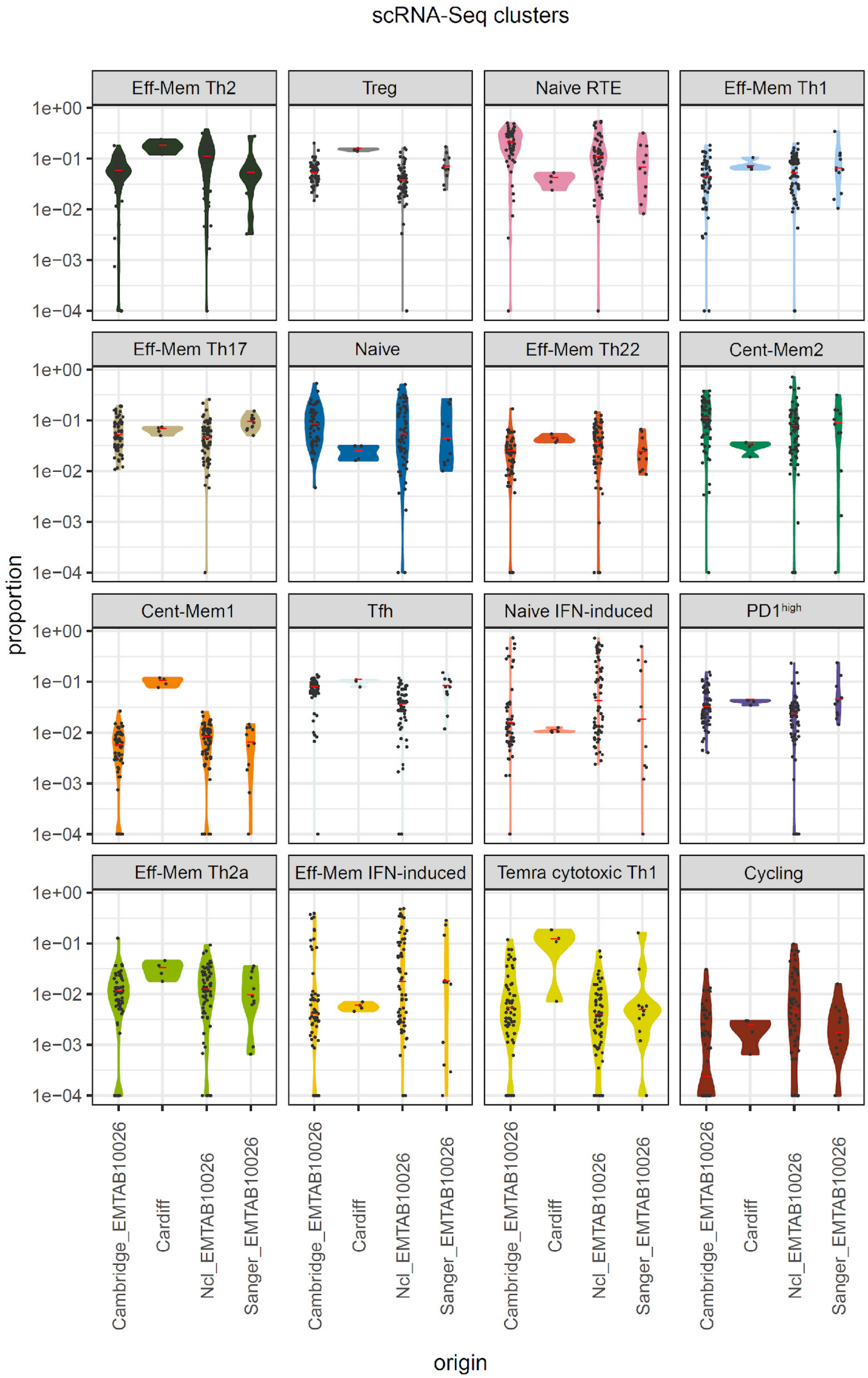

Supplementary Figure 3. scRNA-Seq clusters distribution across studies. Violin plots show proportions of scRNA-Seq Th clusters in individual patients. Patients are grouped according to the study.

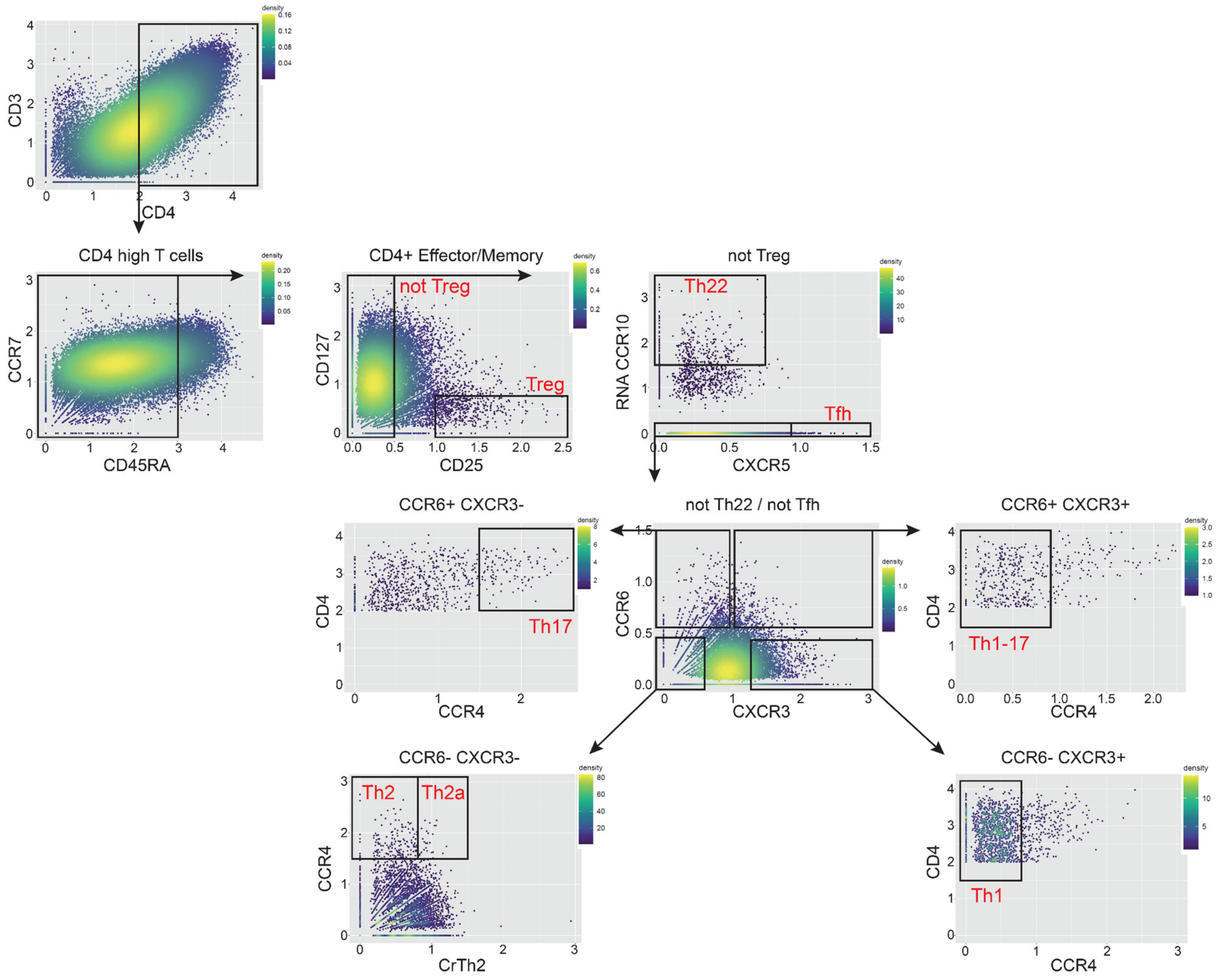

Supplementary Figure 4. *In silico* flow-cytometry-like gating strategy. *In silico* gating with CITE-seq “flow-cytometry-like” biplots corresponding to FACS gating scheme. Only samples with CITE-seq data are included. CCR10 expression is measured by scRNA-Seq due to the absence of CCR10 in the CITE-Seq panel. Color corresponds to the number of neighbor points.

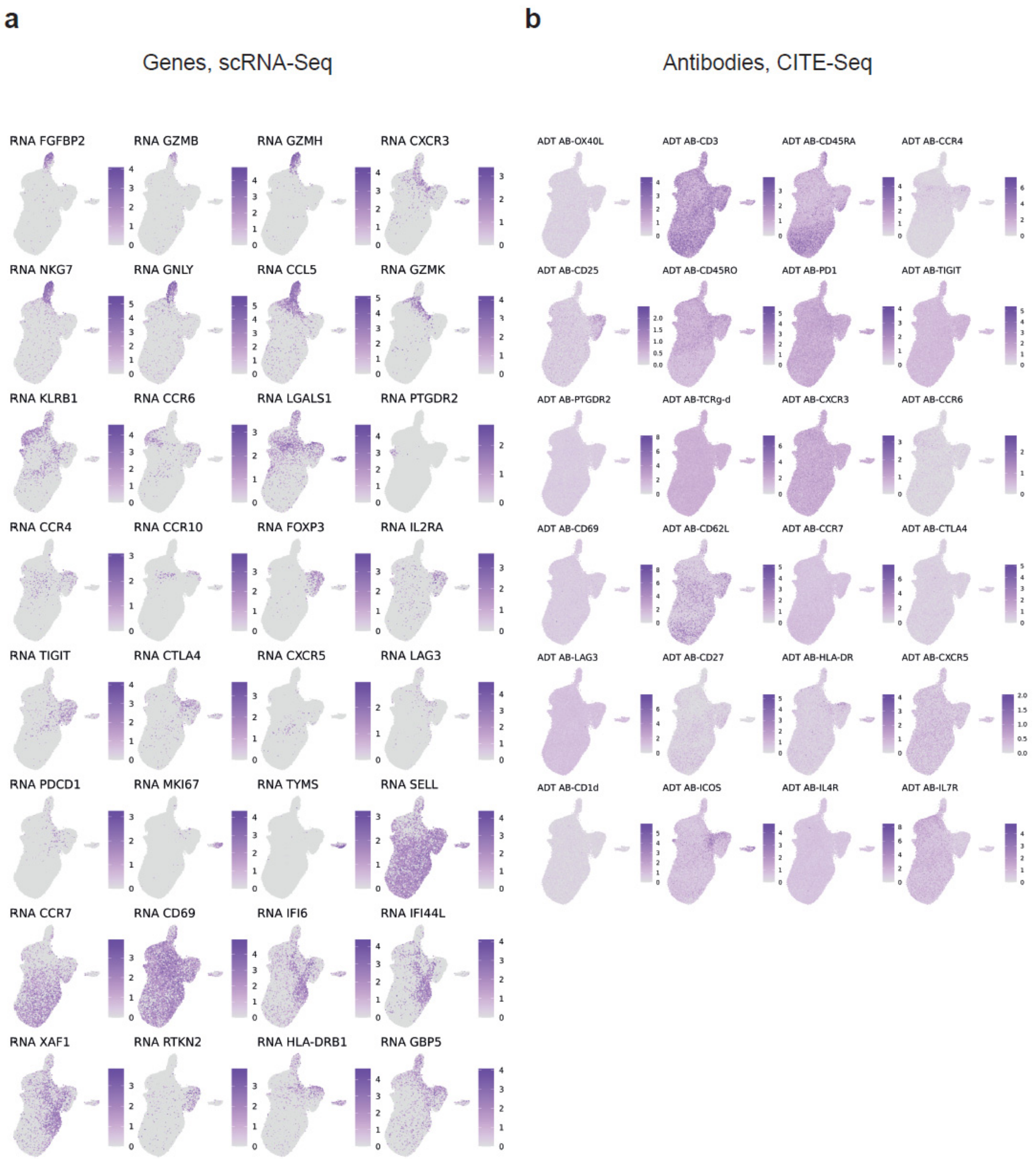

Supplementary Figure 5. Marker gene and protein expression in the reference dataset. a. Marker genes, scRNA-Seq. **b**. Marker proteins, CITE-Seq.

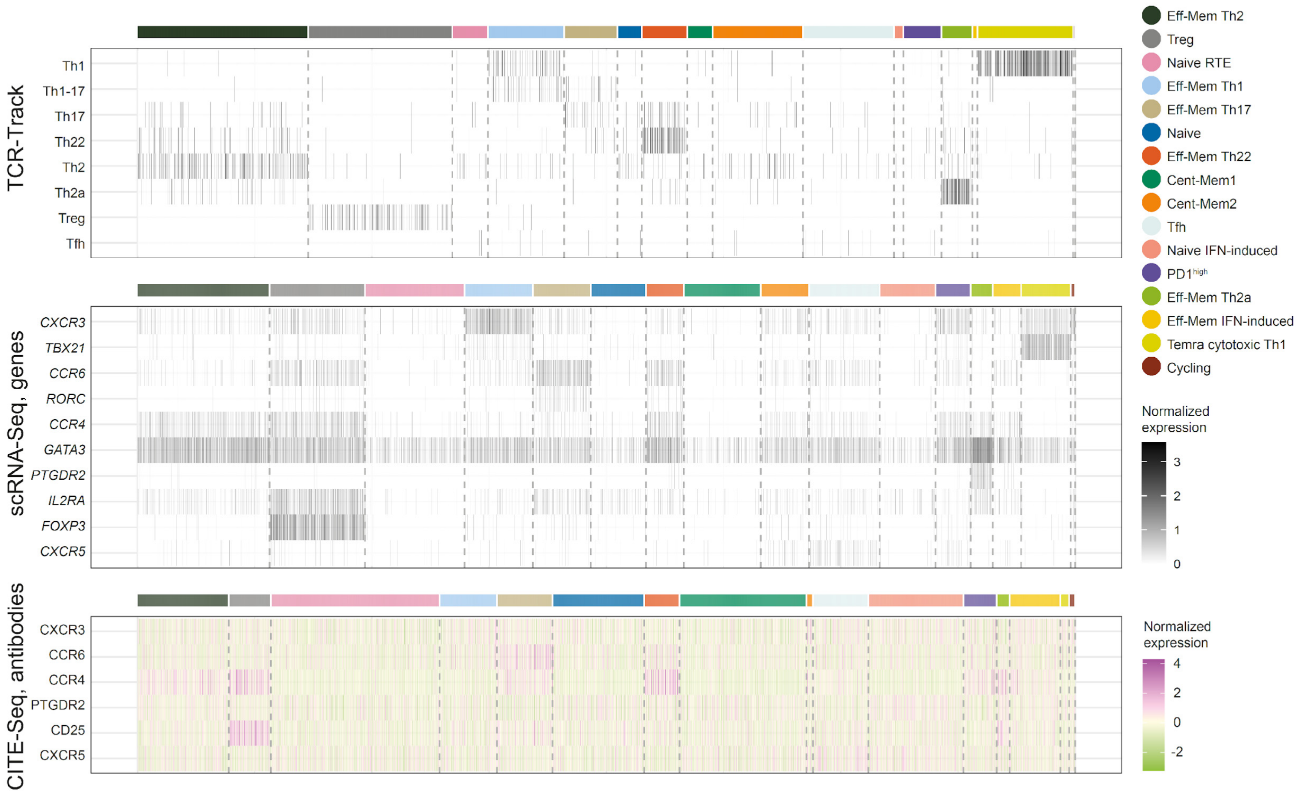

Supplementary Figure 6. Characteristic genes and proteins. Heatmap summarizing the positioning of TCR-Track clonotype-determined subsets, scRNA-Seq expression of characteristic genes, and CITE-Seq signal for the corresponding surface proteins. For normalization, equal numbers of 10,000 scRNA-Seq cells were randomly selected from TCR-Track and CITE-Seq experimental datasets. Each tile of the heatmap represents one cell. The color intensity in the scRNA-Seq plot visualizes the gene expression in a cell (white color depicts zero expression). Protein expression measured by CITE-Seq was Z-scored, and all values exceeding 99.5 percentile were trimmed.

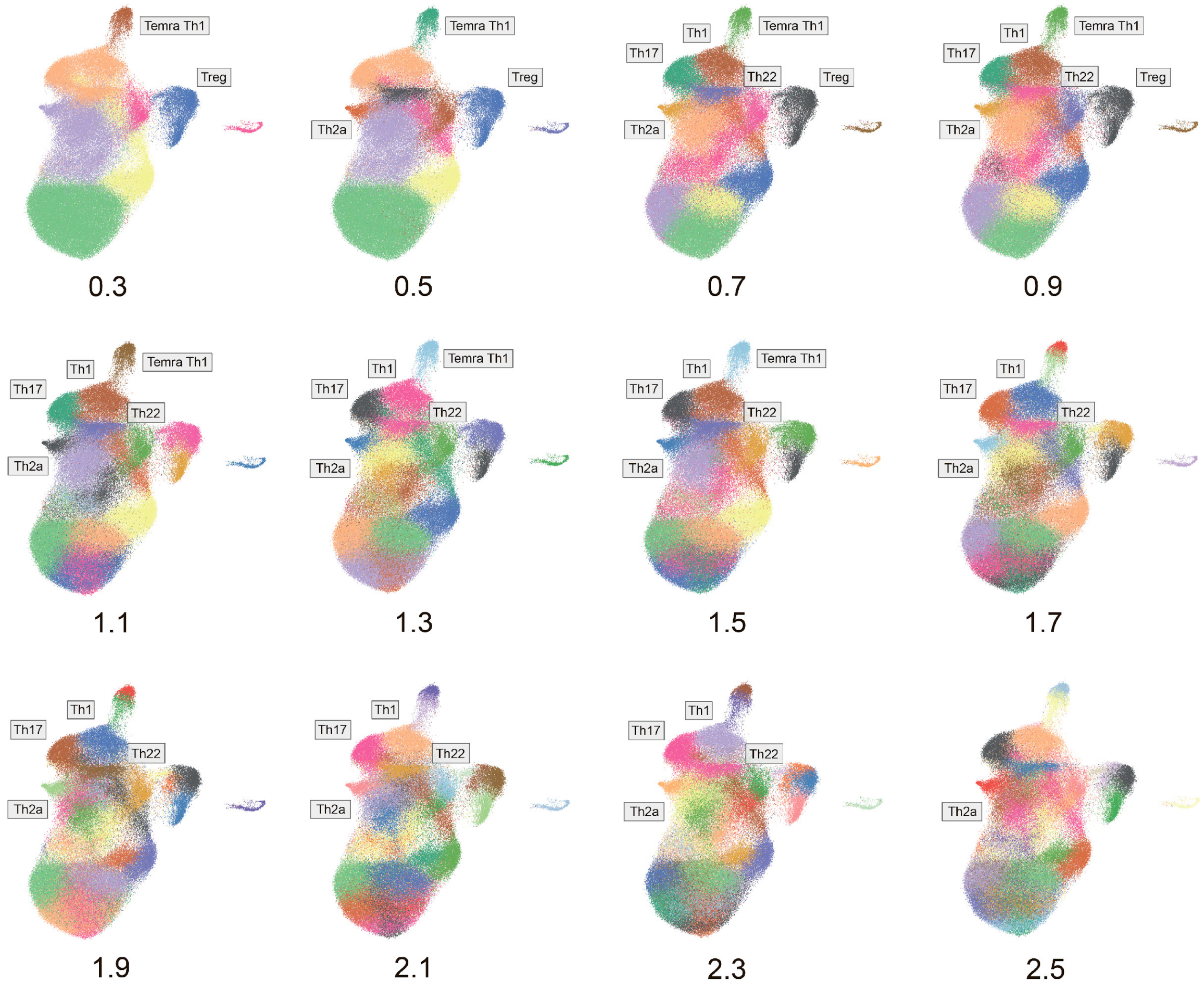

Supplementary Figure 7. Stability of Th programs studied through scRNA-Seq data. UMAP plots built with different clustering resolutions. Th1, Th17, and Th22 clusters are stable and conserved across various resolutions.
